# Psychosocial, psychiatric and work-related risk factors associated with suicide in Ireland: optimised methodological approach of a case-control psychological autopsy study

**DOI:** 10.1186/s12888-019-2249-6

**Published:** 2019-09-06

**Authors:** E. Arensman, C. Larkin, J. McCarthy, S. Leitao, P. Corcoran, E. Williamson, C. McAuliffe, I. J. Perry, E. Griffin, E. M. Cassidy, C. Bradley, N. Kapur, J. Kinahan, A. Cleary, T. Foster, J. Gallagher, K. Malone, A. P. Ramos Costa, B. A. Greiner

**Affiliations:** 1 National Suicide Research Foundation and School of Public Health, College of Medicine and Health, University College Cork, Western Gateway Building, Cork, Ireland; 2 Department of Emergency Medicine, University of Massachusetts Medical School, Worcester, 01655 USA; 3 Diocesan Safeguarding, Kerry, Ireland; 4 School of Public Health, College of Medicine and Health and National Perinatal Epidemiology Centre, Department of Obstetrics and Gynaecology, Cork University Hospital Maternity Hospital, Wilton, Cork, Ireland; 5 National Suicide Research Foundation and School of Public Health, University College Cork, Western Gateway Building, Cork, Ireland; 6 National Suicide Research Foundation, University College Cork, Western Gateway Building, Cork, Ireland; 7 St. Patrick’s Mental Health Services, Cork, Ireland; 8 School of Public Health, College of Medicine and Health, University College Cork, Western Gateway Building, Cork, Ireland; 9 Department of Psychiatry and Neurobehavioural Science, University College Cork, Liaison Psychiatry Service, Cork University Hospital, Cork, Ireland; 10 Department of General Practice, University College Cork, Western Gateway Building, Cork, Ireland; 11 Centre for Mental Health and Safety, University of Manchester and Greater Manchester Mental Health NHS Foundation Trust, Manchester, UK; 12 North Lee Psychiatric Services, Mercy University Hospital, Cork, Ireland; 13 Geary Institute for Public Policy, University College Dublin, Dublin, Ireland; 14 Consultant Psychiatrist, Omagh and Fermanagh, Northern Ireland; 15 School of Public Health, University College Cork, Cork, Ireland; 16 School of Medicine, University College Dublin, Dublin, Ireland; 17 School of Public Health and National Suicide Research Foundation, University College Cork, Cork, Ireland; 18 School of Public Health, College of Medicine and Health, University College Cork, Cork, Ireland

**Keywords:** Suicide, Psychological autopsy, Case-control, Methodology, Psychosocial, Psychiatric, Occupational, High-risk self-harm, Family informants

## Abstract

**Background:**

Suicide has profound effects on families and communities, but is a statistically rare event. Psychological autopsies using a case-control design allow researchers to examine risk factors for suicide, using a variety of sources to detail the psychological and social characteristics of decedents and to compare them to controls. The Suicide Support and Information System Case Control study (SSIS-ACE) aimed to compare psychosocial, psychiatric and work-related risk factors across three groups of subjects: suicide decedents, patients presenting to hospital with a high-risk self-harm episode, and general practice controls.

**Methods:**

The study design includes two inter-related studies; one main case-control study: comparing suicide cases to general practice (GP) controls, and one comparative study: comparing suicide cases to patients presenting with high-risk self-harm. Consecutive cases of suicide and probable suicide are identified through coroners’ registration of deaths in the defined region (Cork City and County, Ireland) and are frequency-matched for age group and gender with GP patient controls recruited from the same GP practice as the deceased. Data sources for suicide cases include coroners’ records, interviews with health care professionals and proxy informants; data sources for GP controls and for high-risk self-harm controls include interviews with control, with proxy informants and with health care professionals. Interviews are semi-structured and consist of quantitative and qualitative parts. The quantitative parts include a range of validated questionnaires addressing psychiatric, psychosocial and occupational factors. The study adopts several methodological innovations, including accessing multiple data sources for suicide cases and controls simultaneously, recruiting proxy informants to examine consistency across sources.

**Conclusions:**

The study allows for the investigation of consistency across different data sources and contributes to the methodological advancement of psychological autopsy research. The study will also inform clinical and public health practice. The comparison between suicide cases and controls will allow investigation of risk and protective factors for suicide more generally, while the comparison with high-risk self-harm patients will help to identify the factors associated specifically with a fatal outcome to a self-harm episode. A further enhancement is the particular focus on specific work-related risk factors for suicide.

## Background

Suicide is a global health concern, with approximately 800,000 persons around the world dying by suicide each year [1]. The causes of suicide are complex. Current evidence-based models emphasise an interaction between pre-existing vulnerability, in particular psychiatric history, previous self-harm, personality factors, family history of suicide, childhood adversity and precipitant stressors, such as significant loss, relationship breakdown, or other psychosocial crises [2–4].

The profound effects of suicidal behaviour are borne by those who survive an act of self-harm, but also by family members, friends, work colleagues, healthcare professionals, and the wider community [5, 6]. Those who are bereaved by suicide endure lasting negative effects on their mental and physical health, and are themselves at increased risk of suicidal behaviour [7–9].

Despite its significant societal impact, suicide is a statistically rare phenomenon (with a global rate of 11 per 100,000). Therefore, one of the most efficient ways of studying the determinants of suicide is to examine risk factors retrospectively using a psychological autopsy approach. This approach is based on the “meticulous collection of data that are likely to help reconstitute the psychosocial environment of individuals who have committed suicide and thus understand better the circumstances of their death” [10]. The approach is useful in assessing psychological characteristics, psychosocial circumstances, health service use and proximal risk or contributing factors associated with suicide, provided that standardised definitions and systematic procedures are used [11, 12].

Psychological autopsy studies have become more widely used in recent years [11, 12], but the potential of this approach has yet to be exploited fully. Some psychological autopsy studies fail to include a control group, and where controls are used, methodological shortcomings include an imbalance of available information between cases and controls and absence of a matched comparison between cases and controls [13, 14]. Recent well-designed psychological autopsy studies are limited to particular groups, such as army soldiers [15], farmers [16] and older people living in rural areas [17] or focussed on specific risks to suicide, such as alcohol use disorder [18]. Well-designed general population studies are scarce. Moreover, few psychological autopsy studies have compared fatal suicidal behaviour to near-fatal suicidal behaviour to determine the factors specifically associated with a fatal outcome, as well as proximal protective factors preventing suicide. Cases of high-risk self-harm appear to share some characteristics with suicide [19–21] but more research is required to elucidate the characteristics of those who survive a near-fatal act, and to explore the role of high suicidal intent in such acts. Finally, the reliability of the information obtained from various sources in psychological autopsy studies, and how these are reconciled in the absence of self-report, is an ongoing methodological issue that requires further testing of empirical data [22]. These methodological issues can be overcome for the most with a thoughtful psychological autopsy study design.

Another consideration in designing a psychological autopsy study is the model that will be used to interpret risk factors for suicidal behaviour. The aetiology of suicide is multi-faceted and includes an array of risk and protective factors. The Integrated Motivational Volitional (IMV) model by O’Connor [3] addresses the transition of suicidal intent to suicidal behaviour while taking into account both individual-level and environmental factors. The IMV model is a diathesis-stress model which specifies components of the pre-motivational, motivational and volitional phases of suicidality, and may therefore provide insight into factors that either increase the risk associated with suicidal behaviour (non-fatal and fatal) or protect effect against suicidal behaviour. The IMV model also allows testing hypotheses on interactions between individual-level (e.g. depression, coping) and environmental (e.g. work-related stress, unemployment) factors.

Although there has been increased recognition of the role of employment and working conditions in suicide during the last global economic recession [23, 24], so far, work-related factors have only been examined in detail in one previous psychological autopsy study [25], which is somewhat outdated, involving cases of suicide in Germany some 15 years ago. However, job stressors have been clearly established as risk factors to mental health and there is also scarce research evidence that they are associated with suicide and suicide attempts, specifically skill level [26], precarious work, lack of social support at work, low control and high job demands [27–30].

The current study follows on from the successful implementation of a psychological autopsy study in Cork, Ireland between 2008 and 2012 [31–34] and aims to address some of the gaps and opportunities outlined above. In keeping with the IMV Model [3], the objective of the current study is to examine the predictive value of specific psychosocial, psychiatric and work-related factors associated with suicide and near-fatal self-harm in line with existing models of suicidal behaviour, and to explore the consistency of information across multiple sources. Risk and protective factors can be identified by comparing three groups: suicide decedents, emergency department patients presenting with high-risk self-harm, and GP patient controls. We conceptualised psychiatric and psychological factors at the level of the individual (e.g. history of self-harm, individual coping, substance abuse, depression), psychosocial factors at the level of the psychological and social environment of the individual (e.g. social support) and work-related factors at the level of the labour market environment (e.g. job loss, high job demands).

The ideal approach to identify potentially causal risk factors for suicide would be a cohort study. However, this design would require a large sample size and resources beyond the scope of the current study, as suicide is a rare event. At a suicide rate of 11 per 100,000, a community-based cohort study would require just over 900,000 participants to observe 100 suicides over a one-year period. For events with such low incidence, a case-control study design is the most pragmatic [35].

The following hypotheses will be tested in the actual study: 1) We hypothesise higher prevalence of unemployment and psychosocial work stressors, including high demands, job insecurity, low control and low social support, among the suicide cases compared with GP controls; 2) We hypothesise moderating effects of factors increasing risk of self-harm and suicide (e.g. level of suicidal intent and planning, access to means) and protective factors (e.g. positive coping and quality of social support); 3) In line with the IMV model [3], we hypothesise that protective factors, such as positive coping, quality of social support, and access to treatment are higher among people who have engaged in high-risk self-harm compared to those who have died by suicide, and 4) We also hypothesise higher levels of these protective factors among the GP controls compared to those who have engaged in high-risk self-harm and those who have died by suicide.

## Methods

### Design

The proposed case-control design involves identifying individuals with and without the outcome of interest and examining exposure to potential risk factors retrospectively. The study design includes two inter-related studies; one main case-control study: comparing suicide cases to GP controls, and one comparative study: comparing suicide cases to patients presenting with high-risk self-harm. Cases were identified through coroners’ records of consecutive inquests of cases of suicide or probable suicide. GP controls were recruited from the same general practices that suicide decedents attended, to control for GP practice variation, and were frequency-matched for age and gender. GP patients were chosen as the control group allowing for a group that resembles the general population and to allow matching on latent variables. By approaching individuals with the support of their GP, we expected to minimise the self-selection bias that can arise when recruiting from the general population.

### Comparisons

**Fig. 1:**
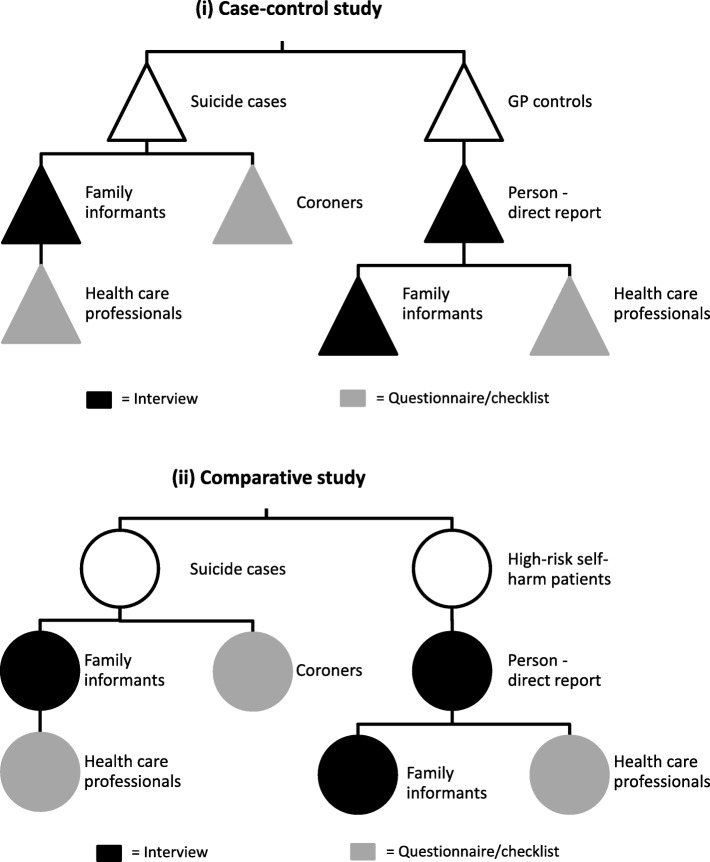
Sources of information for (i) case-control study and (ii) comparative study

This design allows for two sets of comparisons on psychosocial, psychiatric and occupational factors: first, the comparison between suicide cases and controls and second, the comparison between suicide cases and patients with a high-risk self-harm act. This approach allowed us to identify risk factors for suicidal behaviour more generally and suicidal behaviour with a specifically fatal outcome. The study employs a multi-methods approach and builds on the research from the regional Suicide Support and Information System [31, 32]. Several sources are used across the three groups of probands (Fig. 1).

### Inclusion and exclusion criteria

#### Cases of suicide

Consecutive cases of suicide and probable suicide were identified through coroners’ registration of deaths in a defined region (Cork City and County, Ireland) between June 2014 and September 2017. Inclusion criteria for suicide cases comprise: (a) the inquested death having occurred within the Cork City and County coroners’ defined catchment areas, (b) the verdict at conclusion of inquest being that of either ‘suicide’ or ‘open’ or a narrative verdict in which the death is likely to have been a suicide, and (c) the death occurring and having gone to inquest within the time scale of the study. Suicide verdicts are returned by coroners when it has been established beyond a reasonable doubt that a person has taken his/her own life. In order to be considered a probable suicide, the death must have been self-inflicted and there must be evidence to suggest that the deceased intended to cause his/her death. In some cases, the means by which the deceased caused his/her death may clearly suggest that it was a probable suicide. The following can be considered as evidence of a death being a probable suicide [36]: 1) explicit verbal or nonverbal expression of suicide intent, such as verbal statement, handwritten note, unambiguous, and/or recorded statement, e.g. audio tape, email, text and social media; 2) implicit or indirect evidence of intent to die, such as expression of farewell, desire to die, expressions of hopelessness, expressions of great emotional or physical pain or distress and/or history of previous self-harm or suicide threats.

Several sources were used to gather information on suicide cases. These included coroners’ records (autopsy and toxicology reports, police, family and witness statements, documentary evidence such as suicide notes), general practitioner surveys, and family member interviews. To be included as a family informant, the person had to be over the age of 14 years and be sufficiently acquainted with the deceased to provide rich information in relation to the deceased’s life. A potential informant was excluded if they were aged younger than 14 years, if contact was deemed to pose a risk to the safety of the researcher (likely intoxication; history of or potential for violence) or the health & safety of the informant, or where the information obtained was unreliable due to cognitive impairment or severe mental illness.

#### Cases of high-risk self-harm

Consecutive presentations of high-risk self-harm to the two emergency departments in Cork City and County were identified over the same time period. “High-risk self-harm” was defined as intentional self-poisoning or self-injury that involved a combination of a highly lethal method and clinical impression of high suicide intent [37–39]. Inclusion criteria for family informants were that the informant must have been over the age of 14 years and be sufficiently acquainted with the patient to provide rich information in relation to the deceased’s life. The inclusion criteria for informants of high-risk self-harm patients were the same as for family informants of suicide cases. Excluded cases were self-harm presentations that did not meet the criterion for highly lethal self-harm and/or did not have a high level of suicide intent.

#### GP patient controls

Patients from GP practices where suicides cases occurred were identified in Cork City and County over the same time period and were frequency-matched for five-year age-band and gender with the suicide cases. Five potential participants were randomly selected from within the given five-year age-band and gender using the GP patient list; if the first selected patient was uncontactable or unwilling to participate, the next patient was contacted and so on. Personal recruitment via introduction of the research by participants’ GPs has shown to improve response rates and minimise self-selection bias, while the personal contact with the GP has been reported as a participation incentive. Not being approached in person has been reported as a reason for rejecting studies [40].

Included participants were selected patients from GP practices in Cork City and County who were older than 14 years of age. To examine the effect of proxy report, we also recruited a family member or close friend of the GP patient to act as a family informant and complete an interview about the GP patient.

Exclusion criteria for the GP patient and informant were history of high-risk self-harm, being aged younger than 14 years, if contact was deemed to pose a risk to the safety of the researcher (likely intoxication; history of or potential for violence) or the informant, or where the information obtained was unreliable due to cognitive impairment or severe mental illness.

### Procedure

In keeping with the psychological autopsy approach, multiple sources of information for cases and controls were accessed, including coroners’ records, proband interviews (for the GP control patients and cases of high-risk self-harm), family informant interviews, and health care professional questionnaires. Table 1 shows the variables that were obtained from the different sources.

**Table 1:** Overview of variables and measures for the four data sources across the three arms of the study (suicides, high-risk self-harm cases, GP patient controls)

	Coroner’s checklist *(suicide cases only)*	Self-report (semi-structured interviews) *(self-harm patients & GP controls only)*	Family informants *(all three arms)*	Healthcare professionals *(all three arms)*
Cause of death and circumstances; Description of self-harm and circumstances	Description of manner and cause of death Circumstances around suicide (objective intent SIS) Date of death Verdict based on inquest Toxicology results	Description of method of self-harm act Circumstances around self-harm act (objective intent SIS) Circumstances around self-harm act (subjective intent SIS)	Description of manner and cause of death Circumstances around suicide/self- harm act (objective intent SIS)	Description of manner and cause of death Circumstances around suicide/self-harm act (objective intent SIS)
Work-related	Employment status Profession and setting Contractual agreement Employment Sector (NACE)	Job insecurity (COPSOQ) Work-family conflict (COPSOQ) Social community at work (COPSOQ) Job demands (JCQ - short form) Job control (JCQ - short form) Job support (JCQ short form) Employment status Profession and setting Contractual agreement Employment sector (NACE)	Job insecurity (COPSOQ) Work-family conflict (COPSOQ) Social community at work (COPSOQ) Job demands (JCQ - short form) Job control (JCQ - short form) Job support (JCQ short form) Employment status Profession and setting Contractual agreement Employment sector (NACE)	Profession and setting
Psychiatric, psychological	Precipitants - Stressful and traumatic events (short checklist) History of non-fatal suicidal behaviour Suicidal behaviour by role models Family/personal history (esp. abuse/violence) Psychiatric history incl. Psychiatric diagnoses Physical health Alcohol and drug abuse Primary care history Psychiatric treatment (incl. Psychotropic medication) history	Precipitants - Stressful and traumatic events (long checklist) History of non-fatal suicidal behaviour Suicidal behaviour by role models Family/ personal history (exp) Psychiatric history (incl. Views of service) Physical health (diagnosed illnesses, pain, reduction of physical capabilities) Alcohol and drug abuse Primary care history (short) Recent depressive symptoms Impulsivity [DII] Coping [Brief COPE]	Precipitants to death - traumatic events (long checklist) History of non-fatal suicidal behaviour Suicidal behaviour by role models Family/ personal history (exp) Psychiatric history (incl. Views of service) Physical health Alcohol and drug abuse Primary care history (short) Recent depressive symptoms Impulsivity (DII) Coping [Brief COPE]	Precipitants to death-stressful and traumatic events (open question) History of non-fatal suicidal behaviour Family/ personal history (esp. abuse/violence) Psychiatric history incl. Psychiatric diagnoses Physical health Alcohol and drug abuse Primary care history Psychiatric treatment (incl. Psychotropic medication) Recent depressive symptoms
Psychosocial	Life events	Social network [DSSI-10] Life events	Social network [DSSI-10] Life events	Life events

#### Coroners’ records (for suicide cases only)

There were three coroners operating in Cork City and County at the time of the study. A researcher visited the offices of the coroners every 2–4 weeks to review cases that had gone to inquest and were returned with a verdict of suicide or open verdict. Open verdicts were included when they met the criteria for probable suicide set out by Rosenberg [36] as described above. Each case file contained: a report of verdict from the inquest; a police summary of events; statements to police by a range of family members, friends, eyewitnesses, police, healthcare professionals, or others involved with the case; and the post-mortem report including toxicology. Some files also included, where relevant, photographs of the scene of death and medical case notes. All documents were reviewed by the researcher using a checklist derived from the list of variables described in Table 1. There were certain items that were consistently recorded in the case files (e.g. date of birth, marital status, occupation, cause of death, presence of alcohol or drugs in toxicology) while others may not necessarily be recorded as part of the investigation (e.g. presence of mental health problems, precipitant stressors, history of self-harm).

#### Interviews with probands and family informants

Across each arm of the study, information about the proband was sought through face-to-face interviews. The format of the interview was identical across the three arms of the study but the procedures for approaching participants was slightly different.

For the suicide arm, family informants were invited to participate in an interview in the weeks following the inquest. In the first instance, a letter was sent to the next-of-kin using details obtained from the coroners’ files. A follow-up phone-call by the researcher 10 days later focused on facilitation of bereavement support, followed by an invitation to take part in an interview at home or at the research offices.

For cases of high-risk self-harm, the link to the researcher was facilitated by the dedicated crisis nurse service in the two emergency departments in the Cork area after eligible patients were identified via a daily telephone check-in with the clinical staff. After verbal permission was obtained to approach the patient, the researcher introduced the study by telephone or in person where possible. The interview took place in the patient’s home or at the research offices in the weeks after their presentation to hospital. Following completion of that interview, the self-harm patient was asked to identify a family informant who would complete the same interview giving information about the self-harm patient.

GP patients were randomly selected from the practices where the suicides were registered and first contact was made by telephone. Like the high-risk self-harm patients, the GP controls first participated in an interview giving information about themselves and thereafter nominated a family informant to participate in the same interview about the GP control. In order to ensure blinding, the interviews for the proband and the family informant were conducted by different interviewers. Having two informants for the proband in two of the study arms allowed the researchers to examine the reliability of the information obtained from family informants in order to inform the interpretation of data obtained in the suicide arm (where self-report was not possible).

#### Health care professional questionnaires (for all three arms)

As a third source of information, a questionnaire was posted to the healthcare professional (most often the GP but also several mental health professionals) of each proband. This questionnaire covered the variables listed in Table 1. A researcher followed up by telephone with the healthcare professional to respond to any concerns or questions. In the case of the GPs of suicide cases, the researcher also liaised with the GP to gain access to records to allow the selection of a control patient from the same practice.

### Variables measured

In addition to standard sociodemographic variables, the variables measured in the current study include psychosocial, psychiatric and work-related factors together with measures of the circumstances of death with use of well-established validated scales. Table 1 details the sources of data for the causes and circumstances of death and the variables classified into one of the three main areas.

#### Cause of death and circumstances

For the suicides and cases of high-risk self-harm, details around the suicide death and self-harm act were recorded such as method and whether alcohol was consumed as part of the act. Objective suicidal intent (for suicides and self-harm patients) and subjective suicidal intent (for self-harm patients only) were measured using the Beck Suicide Intent Scale [39], a very widely used scale with high internal consistency (alpha = − 0.95 [39]; and consistent factor structure [41]. This scale has also been successfully tested with proxy informants within suicide research: Zhang et al. [22] showed a significant correlation between proxy informants and self-harm patient scores of intent of the self-harm episode, and Conner et al. [42] showed moderate agreement based on intra-class correlation coefficients and no significant difference in mean ratings.

#### Work-related variables

Selected scales of the Copenhagen Psychosocial Questionnaire – Long Version II, 2007 (COPSOQ II) were used, with items relating to “Job insecurity”, “Work family conflict” and “Social Community at Work” [43]. “Psychosocial Demands”, “Decision latitude” (or control) and “Social support” at work were measured using scales from the short version of the Job Content Questionnaire (JCQ) previously developed and applied by Sanne et al. [44]. Bullying and harassment at work were measured through two original items. These scales were piloted to check face validity and applicability to proxy informants. Previous studies using the COPSOQ showed good internal consistency of Alpha = 0.77 for “Job insecurity”, Alpha = 0.80 for “Work family conflict” and Alpha = 0.86 for “Social Community at Work” [43]. Sanne et al. [44] reported internal consistency of Alpha = 0.73 for “Psychosocial Demands”, Alpha = 0.74 for “Decision Latitude” and Alpha = 0.83 for “Social Support”.

The sector of employment was determined by the interviewers applying the Statistical Classification of Economic Activities in the European Community (NACE) which is a standard system used in the European Union to classify industries. Additional questions addressed the sector of employment, employment status, the nature of the employment contract (permanent, fixed-term, occasional, sporadic-hourly) and skill discretion (supervisory versus non-supervisory).

#### Psychiatric and psychological variables

Impulsivity is a well-established risk factor for suicidal behaviour and was measured using the 12-item “Dysfunctional Impulsivity” subscale of the Dickman Impulsivity Inventory [45]. This scale has very good internal consistency (Alpha = 0.84–0.85) and concordance with other self-report measures such as Eysenck’s impulsivity scales [45, 46]. It has been previously reliably applied to proxy informants with ratings just slightly underestimated compared to self-report [22].

Coping was measured using the 28-item Brief COPE [47] with 14 scales with two items each. Carver [42] found good internal validity for the subscales and showed that the brief COPE taps similar factors to the original inventory. Cooper et al. [48] examined the reliability of three composite subscales (emotion-focused, problem-focused, and dysfunctional coping) and also found good internal consistency and test-retest reliability.

Religious and spiritual beliefs are well-established protective factors against suicide [49]. These were measured in the current study using the self-report version of the Royal Free Interview [50]. This scale has been shown to have high internal consistency and good test-retest reliability [50, 51].

#### Psychosocial variables

Social support was measured using the 10-item version of the Duke Social Support Inventory [52] with two subscales measuring social Interaction and social satisfaction. With good internal consistency scores when the total score is used [52], the ratings for proxies have been shown to have acceptable agreement with self-report [22, 42].

Life events in the psychosocial environment were measured by a list based on Brugha et al.’s [53] 12-item List of Threatening Experiences. This questionnaire has previously been used in psychiatric patients and informants and showed good concordance between self-report and informant report. The scale also has high test-retest reliability [54]. Life events include, e.g. death of a partner or close relative and friend, break-up of a relationship, problems with the police or loss of a job. Life events were asked for three different time periods, namely childhood, later in life and past year and rated by the respondent according to highest influence on life.

In order to examine and control for the effect of closeness to the proband and the informant’s affective state, both intimacy (verbal item based on the Scale of Perceived Interpersonal Closeness by Popovic et al. [55] and informant wellbeing (Depression Anxiety and Stress Scale 21-item version by Lovibond & Lovibond [56] were also measured. The latter scale has excellent consistency in clinical and non-clinical samples and good correlations between its subscales and BDI, STAI, and BAI [57].

### Sample size calculations

Based on the primary hypothesis of increased risk of suicide associated with unemployment, 70 suicides and 70 controls would confer 0.8 power to detect an odds ratio of 2.5 as statistically significant for unemployment at 40% prevalence. For continuous variables, each paired-sample of 70 participants would provide sufficient power to detect small effect sizes (e.g. 0.25 standard deviation of paired differences) as statistically significant.

#### Ascertaining concordance across sources

Before investigating differences across the three arms of the study, the concordance of sources within each arm will be examined and the effects of informant characteristics (such as age, gender, and affective state) on concordance will be examined. This will inform decisions around the synthesis of the different sources. These calculations will be based on approaches designed to assess inter-rater reliability. For categorical items obtained from two sources, Cohen’s (weighted) Kappa will be used. For categorical items obtained from more than two sources, Kendall’s coefficient of concordance, W, will be used. For continuous items with two sources, Bland-Altman plots will be used, along with Fisher’s intraclass correlation coefficient.

#### Associations between risk factors and suicidal behaviour

In keeping with the research design, there will be two sets of comparisons: the first between suicide cases and GP controls, and the second between suicide cases and high-risk self-harm patients. Adjusted odds ratios and 95% confidence intervals will be estimated from logistic regression models assessing associations between the work-related, psychosocial and psychiatric factors and suicide or self-harm. The extent to which associations between work-related factors and suicide or high-risk self-harm are mediated via psychopathology and other relevant factors will be estimated by inclusion of covariates in logistic regression models. We will look for evidence of interaction between work-related factors and other exposure variables. All analyses will be adjusted for demographics and prior psychiatric morbidity.

### Ethical considerations

The present study received approval from the Clinical Research Ethics Committee of the Cork University Teaching Hospitals. The study’s procedures, progress and quality were overseen by a multidisciplinary steering group and an advisory group.

The groups of participants in the current study were recognised as vulnerable in various ways. Those who are bereaved by suicide are at higher risk of subsequent suicide, increased risk of required admission to psychiatric care, and increased risk of depression [9]. Those who present to hospital with self-harm are also at increased risk of subsequent suicide [58]. Family history of self-harm is common among self-harm patients [59], so it is possible that family informants in the high-risk self-harm arm may also have a history of self-harm. Even among the GP control arm, up to one-quarter of these patients may have incidental mental health problems [60]. As well as involving vulnerable participants, the study addresses the topic of suicidal behaviour, which fits with the description of a “sensitive” topic [61]. Other topics that were also discussed in the interview can also be considered sensitive, including experiences of abuse, crime, and health conditions.

Given that the interviews were with vulnerable groups and relate to sensitive topics, certain safeguards were included to protect participants. In all arms, researchers were trained to facilitate support for participants where appropriate, particularly in the suicide arm where family members may never have received professional support after their loss. The previous implementation of the Suicide Support and Information System (2008–2012) provided evidence for the potential positive impact of such research participation: the majority of suicide bereaved family members took up the recommended support facilitated by the senior researcher [32]. In that study, family informants who had completed the interviews with the senior researcher consistently reported positive feedback, in particular the benefits of sharing their experiences of loss of their family members with a trained professional [32]. Participants were fully informed of the nature of the project before being asked to participate, including the right to withdraw at any time without prejudice, and written evidence of informed consent was obtained. The interview schedule was structured in a way to maximise acceptability, starting with sociodemographic questions and moving to the characteristics of the suicidal act and adverse life events, through to less sensitive topics such as work-related factors and religiosity, and ending with an assessment of the informant’s wellbeing as a screening for current negative affect. The researchers conducting the interviews were trained to at least Masters level, had previous experience with sensitive interviewing, and received additional training of about 30 h centred on conducting interviews. They consulted with an experienced clinical psychology supervisor after each interview and had peer supervision on a monthly basis. Any concerns around the safety of participants were discussed with the supervisor, with the possibility to link with clinicians where appropriate in the high-risk self-harm and GP arms.

## Discussion

The outlined study has the potential to inform suicide prevention practice and research and to advance the methodology of case-control and psychological autopsy research. The study allows investigation of a broad range of risk factors and protective factors for suicidal behaviour due to its unique design of integrating different data sources (primary health practitioner, coroners and family informants). The study also includes three different groups, suicide cases, high-risk self-harm cases and GP controls, allowing for specific comparisons to be made. The comparison between suicide cases and GP controls allows investigation of risk factors and protective factors for suicide more generally, while the comparison with high-risk self-harm patients will help to identify the factors associated specifically with a fatal outcome to a self-harm episode. A particular methodological strength of the SSIS-ACE case control study design is the matching of suicide cases with controls from the same GP practices, thereby controlling for confounders, such as socio-economic aspects and neighbourhood effects. Moreover, the study design pays careful consideration to the potential effects of proxy report on ascertainment of risk and protective factors.

The comparison between the suicide cases and the high-risk self-harm cases will allow for further examination of the specific factors associated with a fatal outcome after a serious suicidal act and the role of less often researched protective factors. This may be particularly relevant to clinical practice, as one particular contribution of this study will be a more in-depth understanding of the precipitating factors that make a self-harm act more or less likely to be fatal. These groups share many common characteristics, however there is little research on factors other than method lethality that account for survival after a high-risk self-harm act.

A further addition to previous research on the determinants of suicidal behaviour is the current study’s particular focus on work-related risk factors for suicide. There is increasing recognition of working conditions as a potential risk factor for suicidal behaviour but also as an environment for the effective delivery of suicide prevention initiatives [1]. Several large-scale international studies [24, 62, 63], have shown that the global economic recession was associated with an increase in suicide rates, but the importance of specific work-related factors has been neglected. A more in-depth understanding may inform occupational health practice to develop specific work-related mental health and suicide prevention services and health promotion initiatives. The study results may also inform population based public health strategies and evidence-based policy development and information campaigns in occupational settings as demonstrated by the Australian Programme ‘Mates in Construction’, a community-based suicide prevention programme for the construction industry [64].

Although the psychological autopsy method has been used in a variety of studies, its methodical soundness has been questioned [14]. Criticisms include recall bias and information bias, as well as shortcomings with assessing psychiatric diagnoses [65]. However, the current study does not seek to assign psychiatric diagnoses and is particularly suited for investigating the validity of the psychological autopsy method by systematically analysing the consistencies and inconsistencies of responses across different types of informants.

The proposed optimised psychological autopsy methodology represents advancement compared to other psychological autopsy studies. One of the most comprehensive case-control psychological autopsy studies by Schneider and colleagues [25], used information from family informants for both suicide and control cases, in addition to interviews with control participants. However, for the suicide cases no additional sources of information were accessed, such as health care professionals, coroners or forensic doctors, and pro-active facilitation of support was not systematically combined with obtaining information from family informants, a limitation that can also be found in more recent psychological autopsy studies [15–17]. In addition, the optimised psychological autopsy method utilizes two comparison groups (matched GP controls and high-risk self-harm cases). It hereby goes beyond the scope of controlled psychological autopsy studies in suicide research with comparison of two groups only [16, 17, 25, 66, 67] and allows examination of the specific factors associated with a fatal act in comparison to non-fatal but severe self-harm acts. One exception with the use of three groups is the case-control study by Nock et al. [15], conducted with an army population. This study compared, similar to our study, suicide cases with two groups: a group of living controls without suicide ideation and a group of living controls with self-reported suicide ideation. However, the advantage of including a comparison group involving patients who engaged in high-risk self-harm is that this enables determining the factors specifically associated with a fatal outcome, as well as proximal protective factors preventing suicide.

Dissemination of the research outcomes will be conducted at several levels, through peer-reviewed papers, international scientific conferences, a final study report (to be disseminated among key stakeholders on the local, national and international level), and local seminars. It is anticipated that the outcomes of the study will inform public and occupational health interventions and clinical practice to prevent suicide and clinical management of those who attempt suicide.

## Conclusion

Due to its comprehensive nature, the current study and optimised methodological approaches have the potential to inform research methodology and suicide prevention practice across a range of disciplines, including public health, epidemiology, psychiatry, psychology, occupational health and general practice.

## Data Availability

Data and materials are available upon request from the corresponding author. Data are available only upon request due to reasons of data protection and in accordance with the ethical approval of the Clinical Research Ethics Committee of the Cork University Teaching Hospitals.

## Abbreviations

COPSOQ II: Copenhagen Psychosocial Questionnaire – Long Version II
IMV model: Integrated Motivational Volitional model
JCQ: Job Content Questionnaire
NACE: European Industrial Activity Classification
SSIS-ACE: Suicide support and information system – a case-control study
